# Efficacy of intravenous iron for restless legs syndrome—moving beyond monotherapy and into the “real world”

**DOI:** 10.1093/sleep/zsae022

**Published:** 2024-01-23

**Authors:** Elie Matar, Nathaniel S Marshall, Brendon J Yee

**Affiliations:** Centre for Integrated Research and Understanding of Sleep (CIRUS), Woolcock Institute for Medical Research, Sydney NSW, Australia; Central Clinical School, Faculty of Medicine and Health, University of Sydney, Sydney NSW,Australia; Department of Neurology, Royal Prince Alfred Hospital, Sydney Local Health District, Sydney NSW, Australia; Centre for Integrated Research and Understanding of Sleep (CIRUS), Woolcock Institute for Medical Research, Sydney NSW, Australia; Department of Health Sciences, Macquarie University, Sydney NSW, Australia; Centre for Integrated Research and Understanding of Sleep (CIRUS), Woolcock Institute for Medical Research, Sydney NSW, Australia; Central Clinical School, Faculty of Medicine and Health, University of Sydney, Sydney NSW,Australia; Department of Respiratory and Sleep Medicine, Royal Prince Alfred Hospital, Sydney Local Health District, Sydney NSW, Australia

Restless legs syndrome (RLS) is a complex, chronic, and potentially disabling condition which can severely impact sleep, mood, and quality of life [1]. Fortunately, pharmacotherapy is available for RLS, but can be associated with significant side effects and complications. Among the most feared and challenging of these is “augmentation” (worsening of RLS symptoms with respect to intensity, timing, or spread) which is usually seen in the context of long-term dopaminergic treatment. With increased levels of prescribing for RLS [2], there has been a conscious shift in mindset away from simply establishing efficacy to understanding how existing treatment modalities should be optimally deployed [3]. In response, most guidelines adopt an algorithmic approach and endorse oral or intravenous iron replacement as a first-line therapy for RLS [4] based on both RCT evidence [5] and strong biological plausibility from wide ranging methodologies implicating disrupted brain iron homeostasis in RLS development [6–10]. Despite this, iron replacement as first-line or monotherapy for RLS remains limited in clinical practice [11]. This may be due to perceived lack of safety and efficacy, long lead time to effect (as with oral iron replacement which can take weeks or months to replenish iron stores), or logistical and funding considerations restricting access to infusion centers. Furthermore, we have limited guidance on the utility of iron therapies in the more common scenario where a patient may have been established on another RLS therapy, as most previous trials of iron therapy have required a wash-out period of other RLS medications.

In this issue of *SLEEP*, Earley et al. [12] present their multicentre, randomized, placebo-controlled trial which aimed to evaluate the efficacy of intravenous ferric carboxymaltose (FCM). Although efficacy for FCM has been demonstrated in previous trials there were a number of important innovations. Firstly, they recruited symptomatic patients (International RLS Study Group Rating Scale (IRLS) score of ≥15) already taking an alternative RLS monotherapy. Uniquely, they combined the primary intervention (750 mg IV FCM at days 0 and 5) with a planned taper of any existing RLS medication up until day 26, allowing them to consider the effect of iron therapy on reducing existing medication impacts. They also defined a co-primary endpoint for determining efficacy, namely, the change from baseline to day 42 in IRLS total score and the proportion of patients reporting “much improved” or “very much improved” on the investigator’s clinical global impression of change (CGI-I). They stipulated that both endpoints needed to be statistically significant for the trial to be deemed positive.

Patients were randomized to receive FCM (*n* = 105) or placebo (*n* = 104) which makes it the largest trial investigating iron therapy. Only one co-primary endpoint reached significance, being change in IRLS total score (least squares mean difference between the two groups of −3.2 points) favoring intravenous FCM. However, the difference between FCM (35.5%) and placebo groups (28.7%) with positive CGI-I response did not reach formal significance. It is also important to draw attention to the number of patients reaching the primary efficacy assessment at day 42 which may have underestimated the effect of iron therapy. Over the course of the study, patients who had an “intervention” were censored from the analysis following that point. This intervention was defined as an increase in dosage from the RLS medication at study entry, the initiation of new or previous RLS medication, and (likely of most relevance) any patient who had an increase of the RLS medication dose over that reached by the end of the tapering period. At day 42, 82.2% of patients in the FCM group reached this efficacy point, as opposed to only 67.3% of patients in the placebo group. This implied that the FCM enabled more patients to tolerate the tapering of the medications to reach the efficacy assessment. This is also supported by comparing the number of patients requiring an intervention (fewer in the FCM group) and the number of days they lasted without the intervention (greater in those receiving FCM). Although not a prespecified endpoint, these figures suggest a use of iron not just as monotherapy, but as an adjunctive therapy and means to reduce impacts of other pharmacotherapies and potentially their long-term side effects.

The authors remind us that the study was not powered to draw definitive conclusions based on the subgroup analyses; however, there are several interesting observations. Important among these was the difference in response according to augmentation status. Change in IRLS and CGI-I between FCM and placebo groups was greater for those without augmentation than for those with uncertain and definitive augmentation. This suggests that FCM, at least in the context of this protocol, was not as effective in reducing RLS symptoms in those with augmentation. There could be a number of reasons for this, with the simplest being that patients with augmentation did not tolerate tapering as much as those without augmentation and therefore were not as represented in the primary efficacy assessment. Another speculation may relate to a difference in the biology of the two scenarios. For example, RLS symptoms in the augmentation group may be driven by aberrant medication-induced changes in the dopaminergic circuitry downstream of the mechanisms targeted by iron therapies. In this regard, emerging biomarkers such as neuroimaging of brain iron content using quantitative susceptibility mapping [13], may be useful for patient stratification and guiding response of RLS to iron therapies in future trials.

Our observation is that prescribing practices in RLS vary substantially between jurisdictions and even centers. The study recruited almost twice as many participants from European centers as from US centers, and FCM was effective for both co-primary endpoints in the European cohort, but neither were significant in the US cohort. This could be explained by the higher proportion of patients with augmentation in the US group, differences in medications, or different numbers of patients requiring an intervention and being censored prior to the primary efficacy analysis. Whether these features are unique to the study sample, or reflect systemic differences in prescribing practices, patient expectations, or even differences in biology are important to understand before generalizing findings between countries and regions.

The study by Earley et al., is large and examines a cohort of patients with RLS on existing therapies and different degrees of augmentation status. However, the complexity of the trial both in terms of design and co-primary endpoint could have underestimated the efficacy of FCM. There are other cautions including the predominantly ethnically white cohort. The study offers important insights for future trial design. It is important to carefully consider disentangling the effects of weaning medications from the primary intervention (especially if the outcome measure does not explicitly account for medication impacts). However, this protocol does encourage an inclusive approach to future trials, enrolling patients irrespective of existing therapies and augmentation status.

From a clinician’s perspective, this study affirms the efficacy and relative safety of iron therapies and challenges us to consider its use not only early in the patient’s illness, but also as an adjunctive therapy, especially for those patients in whom we wish to reduce the impacts and side effects of other RLS medications. We look forward to more studies moving beyond traditional monotherapy approaches and facing the “real” and oftentimes complex world of RLS management.
